# Improvement of the Concrete Permeability by Using Hydrophilic Blended Additive

**DOI:** 10.3390/ma12152384

**Published:** 2019-07-26

**Authors:** José Luis García Calvo, Mercedes Sánchez Moreno, Pedro Carballosa, Filipe Pedrosa, Fabiano Tavares

**Affiliations:** 1 Eduardo Torroja Institute for Construction Sciences, Consejo Superior de Investigaciones Científicas (CSIC), Serrano Galvache, 4, 28033 Madrid, Spain; 2 Department of Inorganic Chemistry and Chemical Engineering, University of Córdoba (UCO), Campus de Rabanales, 14071 Córdoba, Spain

**Keywords:** permeability-reducing additive, hydrophilic crystalline technology, concrete permeability, BSEM-EDAX microanalysis

## Abstract

Crystalline hydrophilic additives are increasingly used as efficient methods for reducing water permeability in concrete. Their effectiveness in hindering water penetration has been proven in different cementitious materials, although scarce information has been reported concerning their action mechanism. In the present work, the efficacy of a hydrophilic blended crystalline mix (Krystaline Add1) as a water-reducing additive has been confirmed. Furthermore, an extended study about how the presence of the additive influences both the fresh state and the hardened state properties is presented. Finally, characterization techniques such as Mercury Intrusion Porosimetry (MIP), X-ray Powder Diffraction (XRD) and Back-Scattered Scanning Electron Microscopy (BSEM) with Energy Dispersive X-ray analysis (EDAX) have been used for deducing the mechanism of the additive. No significant deleterious influence on the concrete properties due to the addition of the additive has been detected. In fact, the additive seems to have provided a positive influence on the concrete given that a slight reduction in the w/c ratio for similar consistency has been detected, with the subsequent improvement of the compressive strength values. Its effectiveness as a water permeability reducing additive has shown encouraging results having reduced the water permeability by approximately 50% during testing. The action mechanism of the studied additive seems to be related to hydration reactions in the presence of water, producing new solid amorphous phases in the concrete bulk.

## 1. Introduction

Resistance against water permeability is a key parameter related to the durability of concrete structures under hydrostatic service conditions. Different types of chemical admixtures, known as permeability-reducing admixtures (PRAs) [1], are commercially available for improving concrete durability by increasing the watertightness of concrete. According to ACI 212.3R.16 [1], PRAs can be classified according the service conditions of the concrete structure being improved: PRAH, or Permeability-Reducing Admixture for Hydrostatic conditions, and PRAN, or Permeability-Reducing Admixture for Non-hydrostatic conditions. In a structure with non-hydrostatic service conditions, the capillary absorption mechanism should be considered; however, for a concrete structure under hydrostatic conditions, water permeability is the main transport mechanism, and should be taken into account.

Hydrophobic or water repellents admixtures and inert and/or chemically active fillers are proposed as PRANs suitable for damp proofing under non-hydrostatic conditions [2]. Crystalline admixtures can be applied both as PRAN and PRAH [3]. An extended family of crystalline additives are commercially available, and are based on unknown confidential descriptions of their compositions [4]. 

In general, crystalline chemicals are hydrophilic materials possessing the ability to react with cement in the presence of water, increasing the density of calcium silicate hydrates within the concrete and producing pore-blocking precipitates in the capillaries and microcracks [1]. Also, self-healing properties have been reported for these types of admixtures [5,6] even when using low dosages (<1%) [7], although non-conclusive results have been reported [4] and the discussion about their real efficiency is still open. 

Different studies have reported the permeability improvement in concrete when incorporating crystalline admixtures [8,9]. Pzderka and Hájková [8], by applying the standard EN 12390-8 for the water permeability test [10], concluded that the watertightness ability of a concrete with crystalline admixtures (Xypex Admix C-1000 NF in 2% of cement weight) improves with the curing age. However, these authors do not give comparative data with a reference concrete. A 70% efficiency in reducing water penetration has been reported [1] in a concrete with crystalline additives compared with a similar reference without additive. The modified European Standard BS 12390 was used for the testing procedure. 

Zheng et al. recently reported a study [11] focused on the relation between the improvement on macroscopic properties, such as the water permeability, and the modifications taking place at microscopic level in cement grouts containing different additions of crystalline additive. However, no studies on concrete specimens have been found in the literature. 

In the present work, the water-penetration performance of a commercial crystalline admixture, Krystaline Add1, has been assessed. Krystaline Add1 is a blended dry powder admixture based on a proprietary mix of organic and inorganic materials able to create a hydrophilic technology that increases the density of calcium silicate. The microscopic modifications related to the water permeability improvements are discussed in this study.

## 2. Materials and Methods 

The chemical composition of the hydrophilic crystalline additive tested in this study is shown in Table 1. The main oxides present are calcium oxide, silicon oxide and sodium oxide.

The efficacy of the crystalline additive was tested in two conventional concrete mixes. A CEM I 42.5R according to EN 197-1 was used. Two crushed siliceous gravels (12/20 mm and 4/12 mm) and one siliceous sand (0/4 mm) were used as aggregates. In one of the concrete mixes, 1 kg/m^3^ of Krystaline Add1 additive was used, while the other concrete mix was made without this additive. The w/c ratio was changed to obtain the same consistency in both concrete mixes. Table 2 shows the nominal compositions of the two evaluated concretes. 

The fresh state of the fabricated concrete mixes was assessed by measuring the consistency according to EN 13350-2 and the density according to EN 12350-6. Different concrete samples were fabricated in order to evaluate the modifications promoted by the Krystaline Add1 additive in the hardened state of the concretes. Three 100 x 200 mm cylindrical concrete specimens for each age and concrete type were produced, and their compressive strengths were measured at 7 and 28 days according to EN 12390-3 standards. During the 90 day curing process, all samples were submerged in water at 20 °C. Three samples of cylindrical concrete specimens each measuring 150 mm × 300 mm and for each type of concrete were taken for conducting permeability testing according to EN 12390-8. Prior to commencing the permeability testing, the concrete specimens were cured by submerging in water at 20 °C for 90 days. The samples were then put in an apparatus connected to a normal air compressor that ensures that 5 bars compressed air continuously. Each specimen was placed on the apparatus ensuring that the water pressure acts upon the upper face where the watertight seal between apparatus and the concrete specimen is located. Water pressure of 5 bars was then applied for 72 h. The specimens were subsequently removed from the apparatus and subjected to the Brazilian tensile strength test, according to EN 12390-6, in which the specimen was split in half perpendicular to the upper face on which the water pressure was applied. Methylene blue powder was then added to the surface of each sample face by using a brush thus clearly revealing the water penetration depth. The penetration depth was measured in ten different points along the penetration front of each sample size by using a digital caliper. The mean and the maximum water penetration depths were recorded for all concrete samples. 

Additionally, 40 mm × 40 mm × 160 mm prismatic specimens were also cast to evaluate the microstructure of the differing concrete mixes for the purpose of explaining the mechanism used by the Krystaline Add1 additive. Three specimens of each concrete type were placed in a receptacle with a water level that covered up ¾ of the samples and cured for 90 days. Afterwards, some parts of the specimens were cut and analyzed using differing techniques. Samples were taken from the upper side of the specimen (where there was not any water contact, only air contact) and from the lower side (the submerged part of the sample). In all the cases, 1 cm^3^ of each concrete sample was used to measure the total porosity and the pore size distribution by using a Mercury Intrusion Porosimeter (MIP, Micromeritics porosimeter Model 9320, Norcross, GA, USA). X-ray Diffraction patterns (D8 Advance of Bruker, Karlsruhe, Germany) were also recorded in powdered samples at room temperature at intervals of 5° < 2θ < 60°, with a step size of 2θ = 0.01973° and 0.5 s per step. Finally, back-scattering electron microscopy (BSEM) with Energy Dispersive X-ray microanalyses were used. The scanning electron microscope used was a Hitachi S-4800 (Tokyo, Japan) equipped with an energy dispersive analyzer BRUKER 5030 (Hamburg, Germany). The samples were embedded into an epoxy resin, cut, polished and then coated with carbon. The size of the prismatic specimens used for the microstructural evaluations was not the perfect one considering the maximum aggregate size used in the concrete mixes. However, the authors wanted to use the exact same concrete batch in all the studies, and they wanted to obtain samples with a higher specific surface than that obtained with the standardized prisms for concrete samples with the same maximum aggregate size in order to increase the interaction between the water and the cement matrix.

## 3. Results and Discussion

First of all, the modifications promoted by the addition of Krystaline Add1, as it relates to the concrete characteristics, are shown with consideration to both the fresh and the hardened state. Secondly, the mechanism involved in the performance of this additive as it relates to the modifications promoted in the microstructure due to the interaction of the batched concretes with water is assessed.

### 3.1. Modifications to the Concrete Characteristics Due to the Addition of Krystaline Add1 

#### 3.1.1. Modifications to the Fresh State of the Concrete Mix with the Addition of Krystaline Add1

One of the primary significant aspects noted with the addition of the Krystaline Add1 additive when batching fresh concrete is the reduction of the w/c ratio for a similar slump considering the very low content of Krystaline Add1 used. The reference concrete (REF) slump was 8 cm while the Add1 concrete slump was 9 cm indicating an increase in flowability of the Add1 treated concrete over the REF concrete. However, the w/c of the REF concrete was 0.55 while the Add1 concrete was only 0.51, showing a reduction of water for an equal slump. This clearly demonstrates that even though a lower w/c ratio was used (7% less) in the Krystaline Add1 batch, its slump was slightly higher (12.5%). The obtained results indicate a significant filler action by the additive that increases the fluidity of the concrete mix. The increase in fluidity of fresh concrete due to the use of very small powdered particles is well known. For example, lime powder, basalt powder and marble powder are widely used to increase the fluidity of self-compacting concretes and flow-able concretes [12,13,14,15]. Other supplementary cementitious materials with low particle sizes such as fly ash and other powdered industrial by-products can also promote the same performance although with lower efficiency [16,17,18]. It should be noted when dosing the above-mentioned additions for the purpose of increasing concrete fluidity, the dosages used are much higher than the dosage of Krystaline Add1 used in this study, so this additive shows high efficiency in increasing the concrete fluidity.

The density of the fresh concrete mixes was very similar in both cases: 2.35 g/cm^3^ for REF concrete and 2.37 g/cm^3^ for Add1 concrete. Therefore, Krystaline Add1 additive does not generate any significant modification in this parameter.

#### 3.1.2. Modifications to the Hardened State of the Concrete Mix with the Addition of Krystaline Add1

Table 3 shows the compressive strength values measured in the batched samples. The values obtained at each curing age in each concrete type are very similar, thus informing us about the high repeatability of the obtained results. In this sense, Add1 concrete shows higher compressive strength at both curing ages. These results correspond with the lower w/c ratio used in Add1 concrete. In any case, it is important to highlight that the inclusion of the additive under study does not compromise the mechanical performance of the resulting concrete.

**Figure 1:**
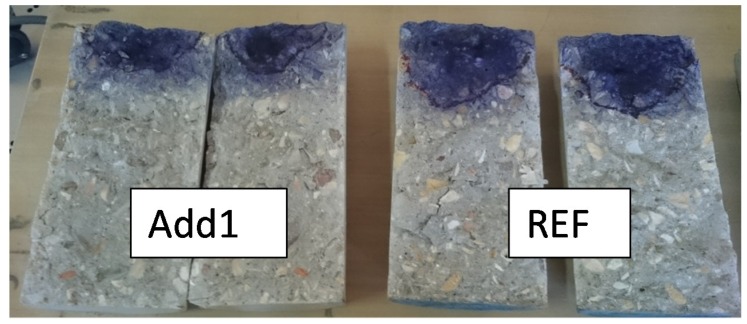
Aspect of two concrete samples after the permeability test and the Brazilian splitting test.

With respect to the water penetration tests, Table 4 shows the mean and the maximum water penetration depths recorded in each case. The values refer to the mean value of three identical samples. A strong decrease (around 50%) of both the mean and the maximum water penetration was detected when using Krystaline Add1 additive compared to the water penetration depths recorded in the reference concrete. The differences between the Add1 concrete and the REF concrete can be clearly appreciated in the image shown in Figure 1, where the water penetration of two concrete samples for both A1 (on the left side) and for the REF sample (on the right side) is marked with methylene blue. Thus, the inclusion of the evaluated additive reduces the water penetration depth, but the lower w/c ratio is achieved in Add1 concrete due to the inclusion of the same additive, which also influences the obtained results. However, the differences obtained in the water penetration depths are too high to be explained only by the decrease of the w/c ratio. In this sense, considering the obtained results, the efficiency of Krystalline Add1 additive in reducing the water penetration into the concrete matrix is quite high. 

### 3.2. Modifications to the Concrete Microstructure Due to the Addition of Krystaline Add1

Once the efficacy of using Krystaline Add1 additive to reduce the water penetration into the concrete matrix had been proven, the mechanisms involved were evaluated by using 40 × 40 × 160 mm^3^ samples that were partially in contact with water for 90 days. Firstly, the porosity microstructure of the concrete samples was assessed. Table 5 shows the total porosity and the pore size distribution. There has not been any significant difference detected between the REF and the Add1 concrete in the total porosity or in the pore size distribution. In fact, the most remarkable aspect was the decrease detected in the total porosity of the submerged sample parts compared to the sample parts with no water contact. In this sense, the decrease detected in the total porosity value of the Add1 concrete after 90 days under water is slightly higher than the one detected in the REF concrete; this total porosity decrease was around 21% in the Add1 concrete, but it was around 19% in the case of the REF concrete. However, this low difference cannot explain the significant decrease detected in the water penetration test in Add1 concrete with respect to REF one and, since this aspect is clearly detected in both concretes, it cannot be only related to the addition of the additive. 

The porosity results obtained with this additive are different from those obtained with other powdered materials that also promote decreased water permeability such as limestone filler. The use of powdered materials with low particle sizes promotes the well-known filler effect. These powdered materials complete the fine portion of the granulometric curve of cement without a relevant increment on water demand, improving the cement packing and blocking the capillary pores. They also constitute nucleation sites of calcium hydroxide crystals and calcium silicate hydrate gels (C-S-H gels) at early hydration ages, accelerating the hydration of clinker particles, especially the C_3_S [19,20,21,22,23]. Thus, the filler effect promotes a decrease in the total porosity or a refinement of the pore size distribution with the subsequent decrease of the concrete water permeability. However, with the powdered crystalline additive used in this study, the decrease in the water permeability should not be related to porosity parameters, so it must be related to modifications in the cement paste composition.

**Figure 2:**
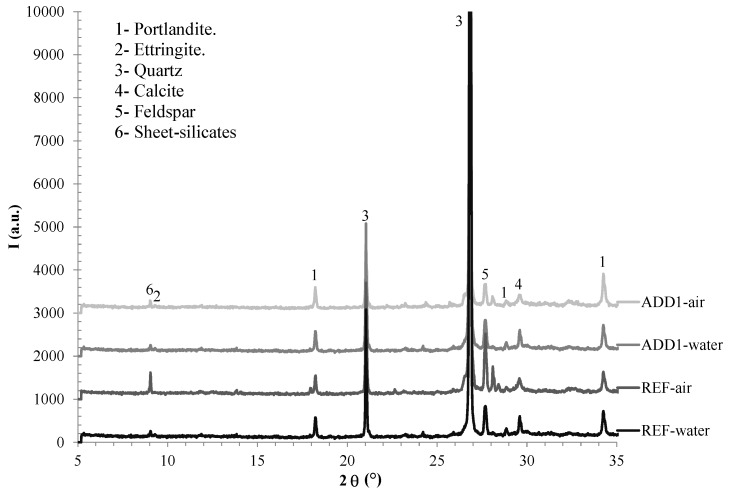
XRD of the concrete samples after 90 days.

Figure 2 shows the XRD obtained in the same samples after 90 days, including both submerged samples and samples with no water contact. The main peaks detected in the diffractograms are related to the siliceous aggregates used and to the main crystalline hydrates of the cement paste (portlandite and ettringite). There has not been any significant difference detected between REF and Add1 concrete in regards to the specimen samples in contact with air (no water contact) or in the specimen samples under water. In fact, the relative intensity of the portlandite and ettringite peaks is very similar in all the cases. Thus, the crystalline components of the cement paste are not influenced by the inclusion of the Krystaline Add1 additive. Moreover, any new crystalline component has not been detected in the Add1 concrete, so the formation of a crystalline phase as responsible for the decrease in the water permeability when the addition Krystaline Add1 additive is rejected.

**Figure 3:**
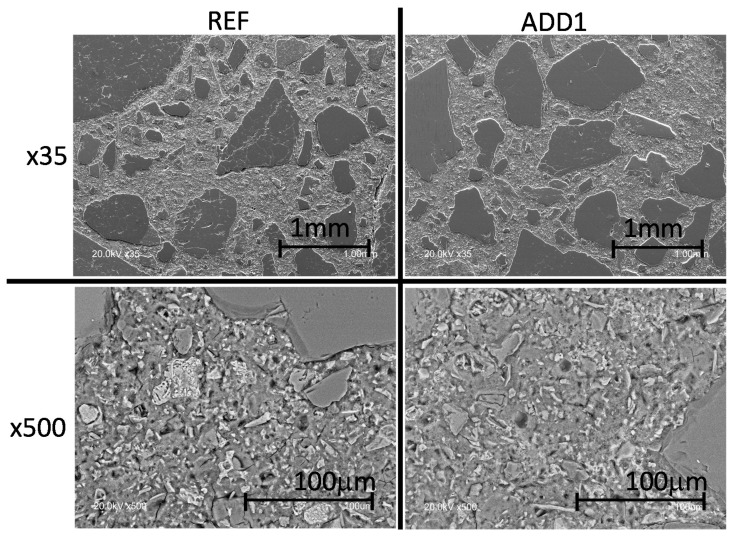
BSEM images: general aspect of REF (left) and Add1 (right) concrete after 90 days underwater.

Therefore, neither the porosity microstructure nor the crystalline component plays a significant role in the decrease of the water permeability detected in Add1 treated concrete. Thus, the possible formation of amorphous phases when adding Krystaline Add1 additive was evaluated by BSEM-EDAX. Figure 3 shows BSEM images of both concretes with different magnifications. Although the MIP results do not show any significant variation between the porosity parameters of both concretes, the BSEM images indicate a more compacted cement paste with the Add1 concrete. This aspect is detected easier in the images with higher magnification, as can be noted in lower half of Figure 3 (×350). In fact, the presence of micropores is more evident in the REF concrete, but due to the smaller size, it may not have been detected by MIP. 

**Figure 4:**
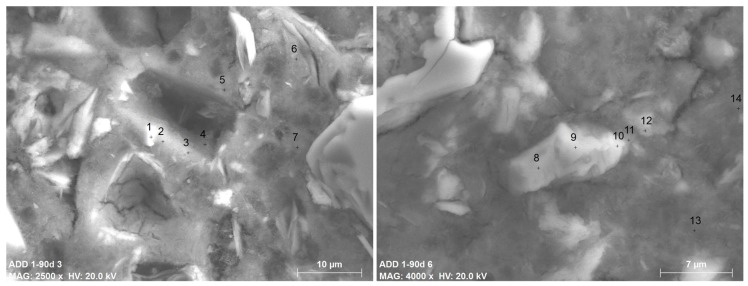
BSEM images of the additive particles and the gel surrounding them in Add1 concrete. Left: ×2500; right: ×4000. The numbers show the exact position of the EDAX shown in Table 6.

The main difference detected between the cement matrix of the REF concrete and the Add1 concrete is the formation of the small phases shown in Figure 4. The numbers specified in both of the BSEM images shown in Figure 4 refer to the EDAX microanalysis shown in Table 6. According to the EDAX microanalyses made, the small particles (with white color in the images) are surrounded by gel/amorphous phases and are mainly composed of CaO, SiO_2_, Na_2_O and Al_2_O_3_, which agree with the chemical composition of the additive used. Therefore, they must be the anhydrous powdered particles of the Krystaline Add1 additive. The hydrated gel surrounding the particles has a different composition with respect to the hydrated gel further from the particles. While the latter has the typical composition of a C-S-H gel of a conventional Portland cement paste, the former has higher Na_2_O and Al_2_O_3_ (and even MgO) contents and lower CaO contents. 

**Figure 5:**
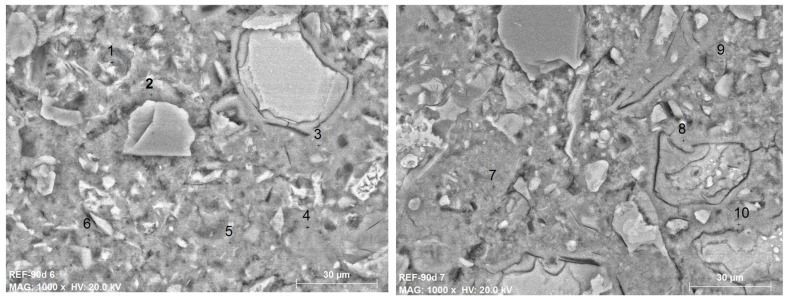
BSEM images ×1000 of the REF concrete. The numbers show the exact position of the EDAX shown in Table 7.

For comparison reasons, Figure 5 shows BSEM images of the REF sample with higher magnification than that of the images of Figure 4. The numbers specified in both of the BSEM images shown in Figure 5 refer to the EDAX microanalysis shown in Table 7. In any case, the anhydrous phases of the additive or the gel/amorphous phases, mainly composed by CaO, SiO_2_, Na_2_O and Al_2_O_3_, are detected in the REF concrete matrix. The EDAX microanalyses of Table 7 show the typical chemical composition of a C-S-H gel of a conventional Portland cement paste. 

According to these results, when water penetrates into the Add1 concrete matrix, the powdered particles from the additive react with the water forming calcium-silicate hydrate gels enriched in Na and Al. Furthermore, possible reactions of these new gels together with the C-S-H gels of the cement pastes cannot be rejected due to the continuing Na content decrease detected from the additive particle to the C-S-H gels. This reaction between both amorphous phases could be indicative of high compatibility of the new hydrates and the cement paste. Then, the formation of Na and Al (even Mg) enriched C-S-H gels must be the mechanism involved in the reduction of the water permeability stimulated by the addition of Krystaline Add1 additive. The formation of these calcium-silica hydrated gels with alkalis and Al in their composition has certain similarities to the hydration products formed from crystalline additives used in self-healing concretes, although with different morphology [24,25,26]. The mentioned hydration products formed from crystalline additives are mainly composed of Ca, Si, Mg, Al and K. When they are formed in the cracks, they have fibrous morphology, but they show orthogonal surfaces when they are formed in the bulk cement paste. These reaction products formed are attributed to hydration reactions involving the crystalline admixtures, which were promoted by the water saturated conditions. Thus, it is possible that the more amorphous morphology of the hydration products formed from Add1 additive with respect to the crystalline hydrates formed when other similar additives are used, facilitates their reaction with the surrounding C-S-H gels. Another different between Add1 additive and the others mentioned above is the absence of sulfur in its composition. When using crystalline additives with more sulfur content (e.g., calcium sulfo-aluminate based agents), the formation of ettringite has been detected when they react with water [24,26]. The formation of ettringite or other calcium sulfo-aluminate hydrate when using Add1 additive is dismissed according to the BSEM and XRD results obtained in the present study. 

Finally, with respect to the EDAX microanalysis made in the C-S-H gel of both concretes, a C/S ratio of 2.10 ± 0.33 is measured in the REF concrete while 1.94 ± 0.29 is measured in the Krystaline Add1 concrete. Considering the size of the microanalysis area, the obtained differences could not be relevant. However, a slight decrease in the C/S ratio in the C-S-H gels when adding Krystaline Add1 additive has not been dismissed, since the results shown in this study clearly identified a decrease of the C/S ratio in the C-S-H gels surrounding the powdered particles of the additive when it reacted with the incoming water. In any event, the differences detected are not very significant. 

Therefore, two types of chemical reactions could explain the mechanisms involved in the effectiveness of the additive evaluated. On one hand, the hydration of the un-hydrated additive particles when water penetrates into the concrete matrix takes part. This hydration of the additive particles generates the formation of amorphous calcium-silica hydrate gels-Na and Al enriched. On the other hand, these new amorphous hydrated gels could also react with the surrounding C-S-H gels previously formed during the cement hydration. This second type of chemical reactions is proposed due to a progressive Na content decrease being detected from the calcium-silica hydrate gels-Na and Al enriched to the C-S-H gels that are close to the formers, that is, the further away from the Na enriched gels, the lower the Na content of the C-S-H gels is and vice versa. In fact, if both types of calcium-silica gels did not react, the inclusion of Na in the C-S-H gels would not have this strong relationship with their distance from the Na enriched gels. Within this regard, the slight decrease of the C/S ratio detected in the C-S-H gels could be related to the introduction of Na into its composition, as previously reported elsewhere, considering C-S-H gels with low C/S ratio [27]. 

## 4. Conclusions

A commercial additive used to reduce water permeability in concrete has been evaluated in the present paper. A complete characterization of the modifications promoted by this additive in the concrete performance has been evaluated and the mechanisms involved in its activity have been analyzed. The use of this additive in the concrete does not result in any loss of performance for concrete and even improves the mechanical properties by reducing the w/c ratio. The efficacy of the additive to reduce the water penetration into the concrete matrix has been also proven. The mechanisms involved in this reduction of the water penetration into the concrete matrix do not seem to be related neither to a filler action nor by the formation of crystalline hydration products, but instead two types of chemical reactions could explain its activity. On one hand, the hydration of the un-hydrated additive particles when water penetrates into the concrete produces amorphous calcium-silica hydrated gels-Na and Al enriched. On the other hand, these new amorphous gels could react with the surrounding C-S-H gels formed during the cement hydration, thus indicating the high compatibility of the new hydrates and the cement paste. 

## Figures and Tables

**Table 1:** Chemical composition (wt.%) of the inorganic additive Krystaline Add1.

Chemical Composition	Al_2_O_3_	Fe_2_O_3_	CaO	SiO_2_	K_2_O	Na_2_O	MgO	SO_3_
**%**	0.95	0.08	17.6	18.6	0.17	26.2	0.21	0.67

**Table 2:** Nominal composition of the fabricated concretes (kg/m^3^).

	REF	Add1
Water	190	180
Cement	350	350
Aggregate 12/20 mm	620	625
Aggregate 4/12 mm	395	400
Sand 0/4 mm	820	830
Additive Add1	-	1

**Table 3:** Compressive strength values obtained in each of the samples tested, mean values and standard deviation (SD).

Sample	Test Age	Compressive Strength. (MPa)	Mean Value	SD
REF	7 days	29.0	27.9	±1.0
		27.9		
		27.0		
	28 days	32.1	30.2	±1.7
		29.7		
		28.9		
Add1	7 days	30.1	31.4	±1.1
		32.2		
		32		
	28 days	36.0	36.1	±1.2
		34.9		
		37.3		

**Table 4:** Mean and maximum water penetration depths (mm) recorded after the penetration of water under pressure test.

		REF	Add1
Mean water penetration depth	Value (mm)	70.7 ± 10.0	36.4 ± 3.38
	% reduction with respect to REF	-	49%
Maximum water penetration depth	Value (mm)	96.8 ± 3.17	53.3 ± 2.89
	% reduction with respect to REF	-	45%

**Table 5:** Total porosity and pore size distribution (%) of the concrete samples after 90 days.

Sample	Experimental Conditions	Total Porosity (%)	Pore Size Distribution (µm, %)			
			∅ > 1	1 > ∅ > 0.05	0.05 > ∅ > 0.01	∅ < 0.01
**REF**	Air contact	9.89	9.86	21.3	39.0	29.8
	Under water	8.01	8.10	9.21	43.5	39.2
**Add1**	Air contact	10.4	11.7	12.4	41.0	29.8
	Under water	8.20	12.3	10.5	39.9	37.3

**Table 6:** EDAX microanalysis specified in the BSEM images of Figure 4 (main oxides, wt.%); Add1 concrete.

EDAX Number	Al_2_O_3_	SiO_2_	SO_3_	CaO	Na_2_O	MgO
**1**	18.2	55.2	1.40	12.2	10.9	1.23
**2**	6.96	35.3	3.96	46.3	1.64	2.20
**3**	5.76	34.5	4.31	46.4	1.20	3.88
**4**	3.29	32.7	1.54	55.8	0.71	1.33
**5**	5.19	29.2	4.98	55.9	0.08	1.13
**6**	8.10	28.8	5.25	53.9	0.24	1.28
**7**	5.96	25.9	6.43	59.8	0.00	0.68
**8**	22.3	61.5	0.23	2.03	13.2	0.45
**9**	22.3	59.9	0.24	2.02	14.8	0.54
**10**	21.6	56.8	1.01	5.89	13.4	0.77
**11**	18.6	46.7	2.59	20.9	8.69	1.01
**12**	14.5	22.8	7.25	50.3	1.44	1.36
**13**	9.69	20.5	5.85	60.3	0.00	0.25
**14**	9.31	21.5	5.78	58.6	0.00	0.51

**Table 7:** EDAX microanalysis specified in the BSEM images of Figure 5 (main oxides, wt.%); REF concrete.

EDAX Number	Al_2_O_3_	SiO_2_	SO_3_	CaO	Na_2_O	MgO
**1**	3.17	26.1	4.76	63.2	ND	0.16
**2**	6.53	29.2	5.45	53.1	1.35	2.40
**3**	3.81	27.6	3.06	62.7	ND	0.30
**4**	3.23	34.1	2.45	56.9	0.42	1.01
**5**	4.95	31.3	4.91	55.1	0.31	1.25
**6**	6.68	22.1	6.60	60.1	0.59	0.25
**7**	3.15	25.0	1.73	64.2	ND	1.51
**8**	1.45	30.1	0.79	62.8	0.60	0.55
**9**	5.06	22.4	2.94	51.4	1.33	0.21
**10**	1.60	28.0	0.80	65.5	ND*	0.54

* ND: Non detected.
